# Inhibition of unintentional extra carriers by Mn valence change for high insulating devices

**DOI:** 10.1038/srep24190

**Published:** 2016-04-12

**Authors:** Daoyou Guo, Peigang Li, Zhenping Wu, Wei Cui, Xiaolong Zhao, Ming Lei, Linghong Li, Weihua Tang

**Affiliations:** 1 Laboratory of Optoelectronics Materials and Devices, School of Science, Beijing University of Posts and Telecommunications, Beijing 100876, China; 2 State Key Laboratory of Information Photonics and Optical Communications, Beijing University of Posts and Telecommunications, Beijing 100876, China; 3 Department of Physics, The State University of New York at Potsdam, Potsdam, New York 13676-2294, USA

## Abstract

For intrinsic oxide semiconductors, oxygen vacancies served as the electron donors have long been, and inevitably still are, attributed as the primary cause of conductivity, making oxide semiconductors seem hard to act as high insulating materials. Meanwhile, the presence of oxygen vacancies often leads to a persistent photoconductivity phenomenon which is not conducive to the practical use in the fast photoelectric response devices. Herein, we propose a possible way to reduce the influence of oxygen vacancies by introducing a valence change doping in the monoclinic *β*-Ga_2_O_3_ epitaxial thin film. The unintentional extra electrons induced by oxygen vacancies can be strongly suppressed by the change valence of the doped Mn ions from +3 to +2. The resistance for the Mn-doped Ga_2_O_3_ increases two orders of magnitude in compared with the pure Ga_2_O_3_. As a result, photodetector based on Mn-doped Ga_2_O_3_ thin films takes on a lower dark current, a higher sensitivity, and a faster photoresponse time, exhibiting a promising candidate using in high performance solar-blind photodetector. The study presents that the intentional doping of Mn may provide a convenient and reliable method of obtaining high insulating thin film in oxide semiconductor for the application of specific device.

The intrinsic semiconductors always exhibit unintentional n- or p- type conductivity induced by native defects such as vacancies or interstitials^1^,^2^, leading to a difficulty in achieving high insulating material and limiting their use in the specific devices such as photodetector, dielectric layer, *etc*. In particular, for oxide semiconductors, the conductivity is often seen to exhibit a pronounced dependence on partial pressure of oxygen during growth^3^,^4^. It seems logical, that this conductivity is related to the presence of oxygen vacancies which served as the electron donors leading to a decrease in resistivity, as has been assumed for many years. In fact, with recent advances in growth techniques, particularly perhaps the advent of novel schemes of molecular beam epitaxy, it has become possible to grow thin films of oxide materials, as required for device applications, with rather high structural quality^5^,^6^,^7^. Notwithstanding this, however, oxygen vacancies have long been^6^,^7^,^8^, commonly and inevitably still are, attributed as the primary cause of conductivity in oxide semiconductors. Meanwhile, the presence of oxygen vacancies in oxides often leads to a persistent photoconductivity phenomenon since the carriers trapped by oxygen vacancies have longer lifetime and drift back to their original states slowly^9^,^10^. It is not conducive to the practical use in the fast photoelectric response devices. Take ZnO based photodetector as an example, the response time would extend to the order of minutes or hours due to the presence of oxygen vacancies^9^,^10^. In this work, we propose a possible way to suppress the unintentional extra carriers which are induced by oxygen vacancies and reduce the influence of oxygen vacancies by introducing a valence change Mn element doping, with a recent hot wide band gap semiconductor material — *β*-gallium oxide (Ga_2_O_3_) using for solar-blind photodetector as an example.

*β*-Ga_2_O_3_, with ~4.9 eV direct band gap and tunable by alloying with Al_2_O_3_ or In_2_O_3_, is particularly suitable for the deep ultraviolet (DUV) photodetector that is blind to wavelengths above 280 nm, which has a vast and ever growing number of military and civil surveillance applications such as missile tracking, fire detection, ozone holes monitoring, chemical/biological analysis, and so on^11^,^12^,^13^. The solar-blind photodetector could detect very weak signals accurately due to the “black background” with the absence of wavelength shorter than 280 nm in solar radiation and artificial lighting. During the past decade, solar-blind photodetectors based on wide band gap semiconductors such as AlGaN, ZnMgO, and diamond, *β*-Ga_2_O_3_ have attracted intensive attentions^11^,^12^,^14^,^15^. But high quality epitaxial AlGaN film is difficult to be prepared due to high growth temperature, single wurtzite phase ZnMgO and diamond are not possible to be used to detect entire deep ultraviolet region due to their mismatched band gaps^11^,^12^. On the other hand, *β-*Ga_2_O_3_ has great thermal and chemical stability, determining its possibility of working at high temperatures and being unaffected even by concentrated acids such as hydrofluoric acid^16^,^17^. Therefore, relatively, *β-*Ga_2_O_3_ is considered as one of the most ideal candidates to fabricate solar-blind photodetector. In our previous work, high epitaxial *β*-Ga_2_O_3_ thin films have been grown via laser molecular beam epitaxy (LMBE) and fabricated metal-semiconductor-metal (MSM) structure using for photodetector, showing an obvious DUV solar-blind photoelectric properties^12^,^18^. At the same time, we also note that oxygen vacancies widely exist inside the gallium oxide thin films and have a significant impact on the photoelectric performance^12^,^19^,^20^. Based on the above, *β*-Ga_2_O_3_ epitaxial thin film using for solar-blind photodetector is an ideal candidate for studying the influence of oxygen vacancies and the suppression of unintentional extra carriers which are induced by oxygen vacancies.

## Results and Discussion

**Figure 1:**
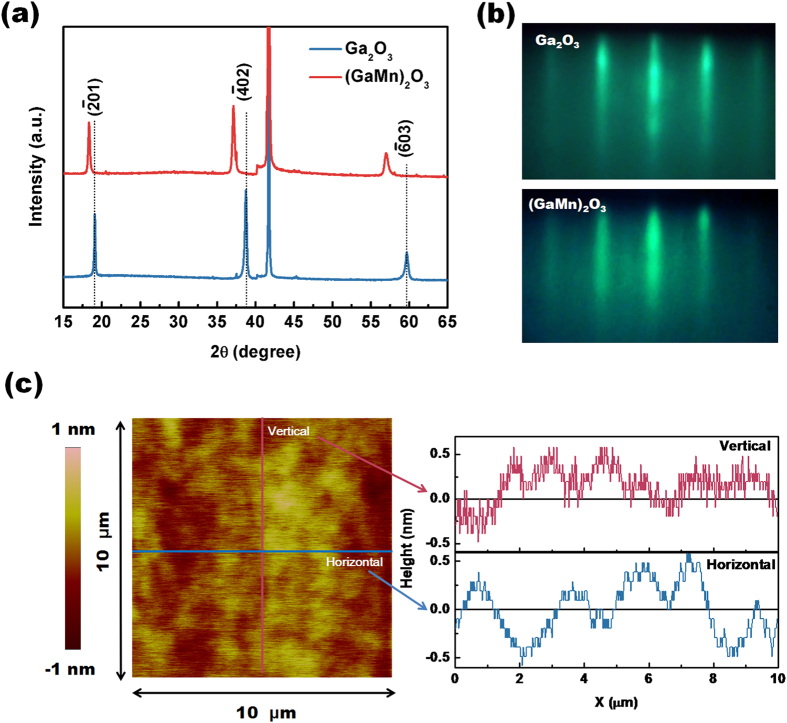
θ-2θ XRD patterns (**a**) and RHEED patterns (**b**) of the *β*-Ga_2_O_3_ and (GaMn)_2_O_3_ epitaxial thin films; (**c**) Surface morphology of the *β*-Ga_2_O_3_ epitaxial thin film.

**Figure 2:**
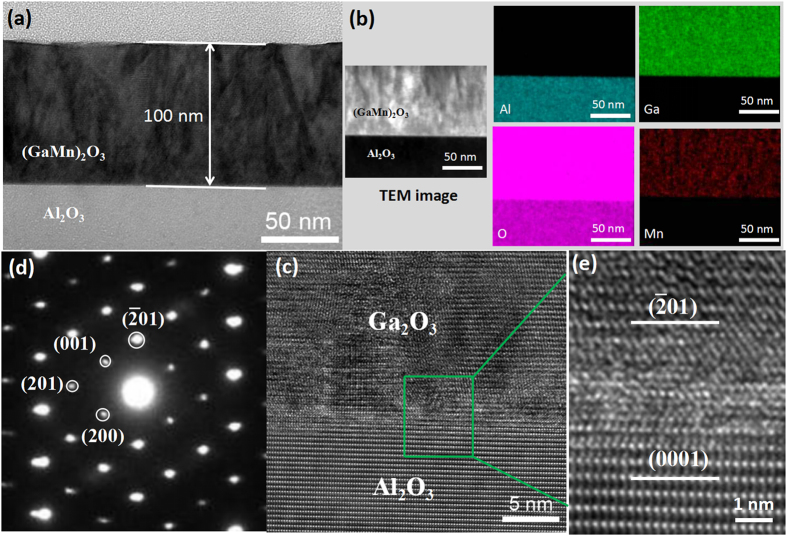
(**a**) Cross-sectional low-magnification TEM bright-field image of the (GaMn)_2_O_3_/Al_2_O_3_ interface; (**b**) TEM-EDX measurement of cross-sectional observation image of the (GaMn)_2_O_3_/Al_2_O_3_ interface, and the composition distributions of Al, Ga, O, and Mn elements drew by different colors; (**c**) Cross-sectional HRTEM image of the Ga_2_O_3_/Al_2_O_3_ interface; (**d**) Selected-area electron-diffraction patterns of Ga_2_O_3_ thin film obtained along [010] aixs; (**e**) High-magnification HRTEM image taken from the Ga_2_O_3_/Al_2_O_3_ interface as marked by a green small pane in (**c**).

Both the undoped and Mn-doped *β-*Ga_2_O_3_ [(GaMn)_2_O_3_] epitaxial thin films were grown on *α*-Al_2_O_3_ (0001) substrate using the LMBE technique, and (GaMn)_2_O_3_ was obtained by alternately depositing Ga_2_O_3_ and Mn thin layers. For both the Ga_2_O_3_ and (GaMn)_2_O_3_ thin film, only the 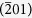 peak and higher order peaks of Ga_2_O_3_ monoclinic *β* phase are presented besides those from the substrate, which indicates that the films are grown with a preferred 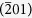 plane orientation [Fig. 1(a)]. For the (GaMn)_2_O_3_ thin film, the positions of these peaks shift to lower 2θ compared to the undoped Ga_2_O_3_ thin film. The shift in the 2θ values indicates an increase of the lattice constants, which can be attributed to the ionic radii difference between Mn and Ga (Mn^2+^, Mn^3+^ and Ga^3+^ ionic radii are 0.83, 0.64 and 0.62 Å, respectively)^21^,^22^. The Mn doping concentration was determined as 28.4 at.% by the X-ray photoelectron spectroscopy (XPS) in the (GaMn)_2_O_3_ thin film. The clear and streaky reflection high-energy electron diffraction (RHEED) patterns suggest that the Ga_2_O_3_ and (GaMn)_2_O_3_ thin films are of single phase with very smooth surfaces [Fig. 1(b)]. The mean surface roughness of the Ga_2_O_3_ epitaxial thin film is 0.239 nm over 10 × 10 *μ*m^2^ scanning area [Fig. 1(c)]. Both the Ga_2_O_3_ and (GaMn)_2_O_3_ thin films have uniform and similar thickness of about 100 nm. Figure 2(a) shows the low-magnification bright-field transmission electron microscope (TEM) image of the (GaMn)_2_O_3_ thin film growth on Al_2_O_3_ substrate. The elemental composition mapping of cross-sectional observation of the (GaMn)_2_O_3_/Al_2_O_3_ interface was obtained to investigate the composition distributions in the (GaMn)_2_O_3_ thin film [Fig. 2(b)]. From the analysis results, Ga and Mn metals are distributed uniformly in the film and there are no noticeable phase separating areas. The cross-sectional elemental mapping was conducted over 10 places; thereby no clear separated phase or metal agglomerated areas were confirmed. It is consistent with the secondary ion mass spectrometry (SIMS) depth profiling in our previous report^23^, indicating that Mn is actually uniformly distributed. The interface microstructure between the Ga_2_O_3_ film and the Al_2_O_3_ substrate were also explored [Fig. 2(c)]. *β* phase of Ga_2_O_3_ has been further confirmed by the selected-area electron diffraction patterns of Ga_2_O_3_ thin film along [010] aixs [Fig. 2(d)]^24^. The high-resolution transmission electron microscope (HRTEM) image shows a sharp interface of the Ga_2_O_3_ and Al_2_O_3_ without any precipitation [Fig. 2(e)]. Meanwhile, it clearly indicates the orientation relationship of 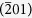 *β*-Ga_2_O_3_//(0001) Al_2_O_3_ and confirms the epitaxial growth of the film. More detailed epitaxial relationship between 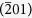 *β*-Ga_2_O_3_ and (0001) Al_2_O_3_ was described in the Supplementary Information.

**Figure 3:**
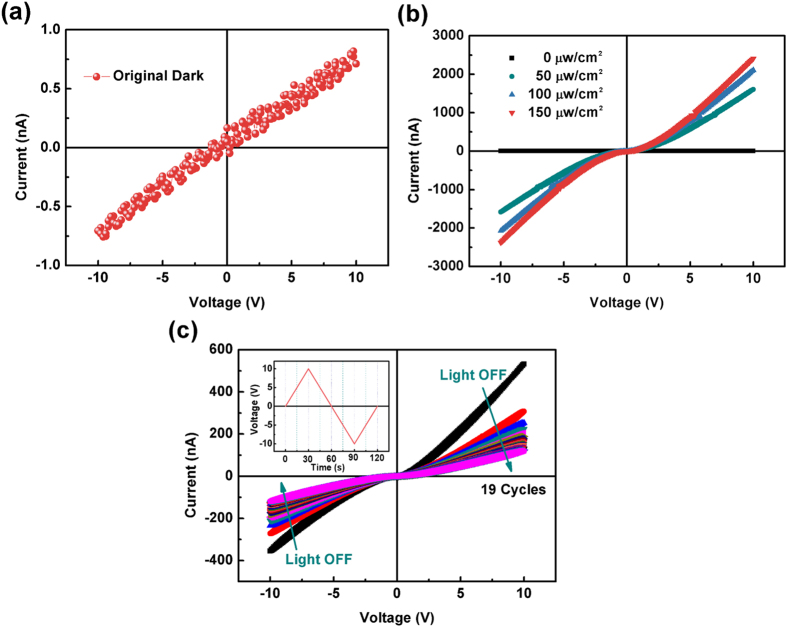
(**a**) the fresh dark *I-V* characteristic curve of the Ga_2_O_3_ photodetector; (**b**) *I-V* curves of the Ga_2_O_3_ photodetector in dark and under 254 nm light with varied optical input power; (**c**) the consecutive sweeps of *I-V* curves of the Ga_2_O_3_ photodetector when the illumination turns off, and the change of sweep voltage is depicted in the upper left inset.

**Figure 4:**
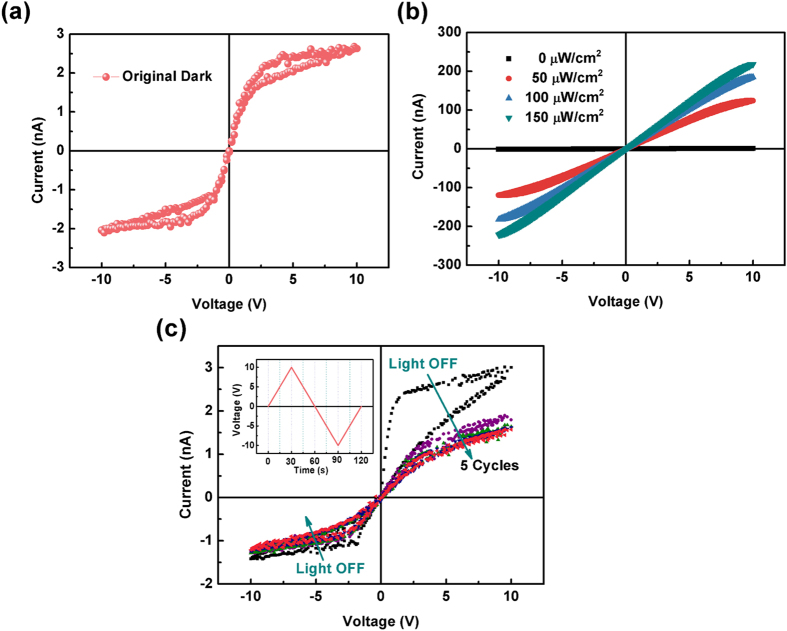
(**a**) the fresh dark *I-V* characteristic curve of the (GaMn)_2_O_3_ photodetector; (**b**) *I-V* curves of the (GaMn)_2_O_3_ photodetector in dark and under 254 nm light with varied optical input power; (**c**) the consecutive sweeps of *I-V* curves of the (GaMn)_2_O_3_ photodetector when the illumination turns off, and the change of sweep voltage is depicted in the upper left inset.

To construct a MSM photodetector, radio frequency magnetron sputtering technique was used to deposit four pairs of Au/Ti interdigital electrode on the epitaxial thin films using a shadow mask [Supplementary Fig. S1]. Figures 3(a) and 4(a) show the fresh dark current-voltage (*I-V*) characteristic curves of the Ga_2_O_3_ and (GaMn)_2_O_3_ thin films based MSM photodetectors respectively. For the Ga_2_O_3_ thin film, the *I-V* curve is linear with a large resistance of 12.5 GΩ. The fresh dark current is about 0.8 nA at the voltage of 10 V [Fig. 3(a)]. While for the (GaMn)_2_O_3_ thin film, the fresh dark current increases quickly from 0 V to 1.3 V (−1.3 V) and then increase slightly as the voltage continually increases to 10 V (−10 V), exhibiting an nonlinear behavior [Fig. 4(a)]. When the photodetectors are exposed to the 254 nm light, the slopes of *I-V* curves show a sharp increase for both the Ga_2_O_3_ and (GaMn)_2_O_3_ thin films [Figs 3(b) and 4(b)]. Meanwhile, the photocurrent increases with the increase of the light intensity. However, when the illumination turns off, the decay process of photocurrent is different for the two devices. The consecutive sweeps of dark *I-V* curves of the Ga_2_O_3_ photodetector after the 254 nm light turns off is shown in Fig. 3(c), and the change of sweep voltage is depicted in the inset. The current *vs* voltage decreases quickly during the several initial *I-V* sweeps and then decrease slightly. Nevertheless, the tiny fresh dark current cannot be obtained again. The dark current is about 120 nA at the voltage of 10 V after turning off the 254 nm light for 24 hours, which increases by about 150 times compared to the fresh dark current [Supplementary Fig. S2]. For the (GaMn)_2_O_3_ photodetector, the dark current can back to the original value in the half circle of the first *I-V* sweep [Fig. 4(c)]. The final dark current after 254 nm illumination is about 1.2 nA at the voltage of 10 V.

**Figure 5:**
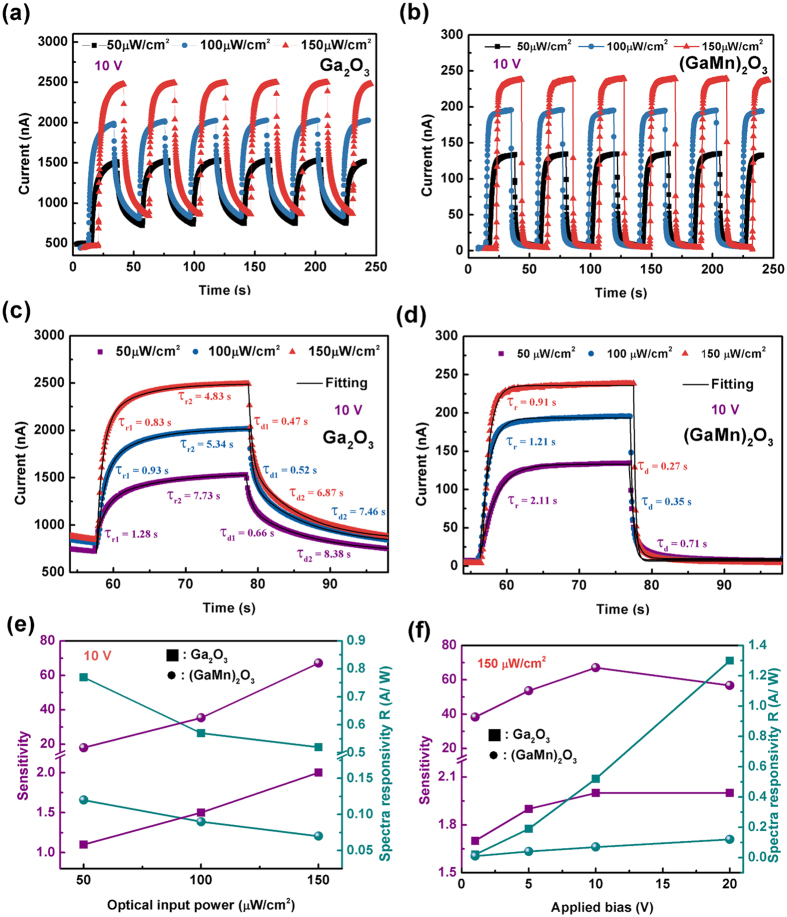
Time-dependent photoresponse of the Ga_2_O_3_ (**a**) and (GaMn)_2_O_3_ (**b**) photodetectors to 254 nm UV light illumination with varied optical input power (50, 100, 150 μW/cm^2^) by on/off switching at 10 V, and (**c**,**d**) are the corresponding fitted curve for the current rise and decay process of (**a**,**b**) respectively. The sensitivity and spectra responsivity (*R*) of the photodetectors with varied optical input power (**e**) and varied applied bias (**f**).

To further evaluate the performance of the Ga_2_O_3_ and (GaMn)_2_O_3_ photodetectors comparatively, the time-dependent photoresponse to DUV illumination were investigated. Figure 5(a,b) show the time-dependent photoresponse of the Ga_2_O_3_ and (GaMn)_2_O_3_ photodetectors to 254 nm UV light illumination with varied optical input power (50, 100, 150 μW/cm^2^) by on/off switching at 10 V respectively. The illumination current increases with increasing incident optical power. For example, for the Ga_2_O_3_ photodetector, the illumination current is 1531.2, 2018.1, and 2490.6 nA for the optical power of 50, 100, and 150 μW/cm^2^, respectively. Meanwhile, for the fixed optical power, both the illumination and dark currents increase with the increase of applied bias [Supplementary Fig. S3(a,b)]. However, there are also some obvious differences between two devices. For the Ga_2_O_3_ photodetector, the current increases from approximately 465.6 nA of original dark current to a non-stable value of approximately 2490.6 nA of illumination current with an optical input power of 150 μW/cm^2^ at 10 V [Fig. 5(a)]. However, the recovery time is extremely long after the light is turned off. Such slow recovery should be attributed to the electron-hole trapping states, which would prevent charge-carrier recombination. And the original dark current cannot be obtained with a duration time of 20 s after turning off the 254 nm light. For the (GaMn)_2_O_3_ photodetector, the dark current is approximately 3.1 nA at 10 V, which is low and favorable for practical detectors. Under 254 nm light with an optical input power of 150 μW/cm^2^ illuminations, the current instantaneously increases to a stable value of approximately 238.5 nA. When the light turns off, the current decreases rapidly down to 3.5 nA, which is quite close to the initial dark value.

The sensitivity, the spectra responsivity (*R*_*λ*_) and the external quantum efficiency (*EQE*) are the key parameters to evaluate the performance of a photodetector (the definition of these parameters please see Supplementary Information)^25^,^26^,^27^. The larger values of sensitivity, *R*_*λ*_ and *EQE* – the higher performance a photodetector has. These the parameters for our two photodetectors are listed in Table 1. The sensitivity increases with the increase of incident optical power, while it increases firstly and then decreases with increasing applied bias [Fig. 5(e,f)]. Meanwhile, the *R*_*λ*_ and *EQE* values increase with the increase of applied bias while decrease with increasing incident optical power. The sensitivity of the (GaMn)_2_O_3_ photodetector is significant superior to that of the Ga_2_O_3_ photodetector due to its tiny dark current. The maximum sensitivity of 67.1 was obtained at 10 V with an optical input power of 150 μW/cm^2^ in the (GaMn)_2_O_3_ photodetector. The band gap for 28.4 at.% Mn doping Ga_2_O_3_ thin film can be estimated to about 4.75 eV, which has a bit red shift compared to undoped Ga_2_O_3_ (4.92 eV)^23^. The light absorption to 254 nm UV light for undoped Ga_2_O_3_ thin film is a bit stronger than Mn-doped one, which contributes to a higher sensitivity. However, the Ga_2_O_3_ photodetector exhibits the bigger *R*_*λ*_ and *EQE* values than the (GaMn)_2_O_3_ photodetector. Herein, the maximum *R*_*λ*_ value of 1.30 A/W was obtained at 20 V with an optical input power of 150 μW/cm^2^ in the Ga_2_O_3_ photodetector, which corresponds to an *EQE* ~634%.

Another important parameter for UV photodector is response time. A bi-exponential relaxation equation was used to analyze quantitatively the current rise and decay process of two devices (please see Supplementary Information). As shown in Fig. 5(c,d) and [Supplementary Fig. S3(c,d)], the photoresponse processes are well fitted. *τ*_*r*_ and *τ*_*d*_ are the time constants for the rise edge and decay edges respectively. We note that both the current rise and decay processes consist of two components with a fast-response component and a slow-response component for the Ga_2_O_3_ photodetector, while there is only a fast-response component for the (GaMn)_2_O_3_ photodetector. Generally, the fast-response component can be attributed to the rapid change of carrier concentration as soon as the light is turned on/off, while the slow-response component is caused by the carrier trapping/releasing owing to the existence of defects in *β*-Ga_2_O_3_ thin films such as oxygen vacancies. For example, for the 254 nm illumination with an optical input power of 150 μW/cm^2^ at 10 V, the decay process is rapid with a *τ*_d_ of 0.28 s for the (GaMn)_2_O_3_ photodetector, while the decay process of the Ga_2_O_3_ photodetector is slow which consists of two components (*τ*_d1_ = 0.47 s, *τ*_d2_ = 6.87 s). The (GaMn)_2_O_3_ photodetector presents a much faster response speed to light than that of the Ga_2_O_3_ photodetector.

**Figure 6:**
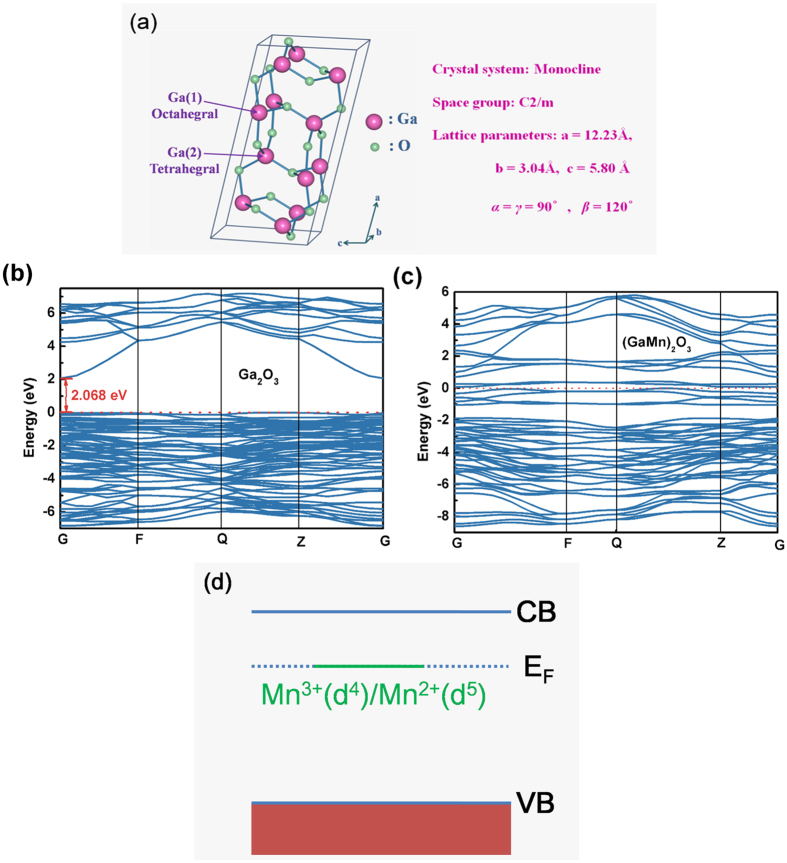
(**a**) The conventional unit cell of monoclinic *β*-Ga_2_O_3_; Band structure plots of pure *β*-Ga_2_O_3_ (**b**) and Mn replacing two adjacent octahedral sites in an conventional unit cell with a dopant concentration of 25% (**c**,**d**) Schematic diagram showing mid-gap Fermi level near the Mn^2+^/Mn^3+^ transition level.

To understand the impact of the Mn dopants on the conducting properties, the crystal structure was built [Fig. 6(a)]^28^, and the electronic structure was calculated with density functional theory (DFT). Figure 6(b,c) shows the electronic structure of undoped and Mn-doped *β-*Ga_2_O_3_ respectively. The Fermi level was set at zero-point of energy. For the calculation of band structure of undoped *β-*Ga_2_O_3_, a 1 × 2 × 1 supercell doubling the monoclinic unit along the *b* direction was modeled. The calculated width of the band gap is 2.068 eV (uncorrected), which is less than half the experimental value of 4.9 eV. This is because DFT theory is based on the ground state theoretically, resulting in that the exchange-correlation potential between the excited electronic has been underestimated. Before the calculation of band structure of Mn-doped *β-*Ga_2_O_3_, the site occupancy of the Mn dopants was investigated by the total energy density functional theory calculation. With the Mn doping concentration of 28.4 at.%, two Mn ions replacing with two Ga ions in a conventional unit cell with a dopant concentration of 25 at.% was modeled. Comparing the total energy, those two Mn ions substituting two adjacent octahedral sites is found energetically stable, and the electronic band structure is given in Fig. 6(c). Relative to undoped *β-*Ga_2_O_3_, there are six new bands within the band gap occupying energies. For the neutral cell, which corresponds to 3+ valence for the Mn, two thirds of these new states are occupied, and the other unoccupied; that is, the Fermi level is located in these defect states. Mn^3+^ is amphoteric since it can accept and donate an electron if the Fermi level crosses the Mn^2+^/Mn^3+^ acceptor or the Mn^3+^/Mn^4+^ donor level^29^,^30^. In our previous report, two valence states of Mn ions (Mn^2+^/Mn^3+^) were observed in our (GaMn)_2_O_3_ thin films by using XPS^23^. Therefore, the Fermi level should be located within *kT* of the Mn^2+^/Mn^3+^ transition level, which is depicted schematically in Fig. 6(c). In this model, if the Fermi level is higher, additional electrons would enter the Mn *d*-shell, and thus decreasing the valence to 2+^29^,^30^,^31^. In other words, if Mn doping is the dominant defect in *β-*Ga_2_O_3_, the Fermi level will be pinned close to the Mn^2+^/Mn^3+^ transfer level. In our undoped *β-*Ga_2_O_3_ thin film, oxygen vacancies always existed as the donor-type defects^23^, attributed as the primary origin of intrinsic n-type conductivity. For the Mn doping, the extra electrons introduced by oxygen vacancies would enter the Mn *d*-shell, and the neutral acceptors (Mn^3+^) are converted to negatively charged acceptors (Mn^2+^). The change of Mn valence suppresses the unintentional extra carriers, and the Mn ion acts as the “carrier killer”. Therefore, the (GaMn)_2_O_3_ thin film has a higher resistivity compared to the undoped Ga_2_O_3_, leading to a lower dark current in the application of photodetector. At the same time, the oxygen vacancies are usually acted as the carrier trapping/releasing centers in the photodetector. Under the illumination of 254 nm light, some of the photogenerated carriers are captured by the trapping states of oxygen vacancies. When the illumination is turned off, these carriers captured by the oxygen vacancies would be released and recombined. Generally, traps in a wide band gap semiconductor are extremely deep. The time constant of the transient decay is governed by the depth of these traps and can be very long, and this process is responsible for the slow-response component. For the as-grown undoped Ga_2_O_3_ thin films, the II peak of O 1s XPS which is usually associated with the oxygen vacancy is obvious^23^. While for the (GaMn)_2_O_3_ thin film, the II peak of O 1s is highly suppressed, indicating that the oxygen vacancy concentration decreased markedly [Supplementary Fig. S4]. The presence of many oxygen vacancies prevents carriers’ recombination and causes the slow recovery time for pure *β-*Ga_2_O_3_ thin film. And the reduction of oxygen vacancies concentration by Mn doping contributes to a faster response speed to light for (GaMn)_2_O_3_ photodetector.

In conclusion, we propose a possible way to suppress the unintentional extra carriers and reduce the influence of oxygen vacancies in *β-*Ga_2_O_3_ thin film by introducing a valence change element Mn doping. The high insulating monoclinic *β-*Ga_2_O_3_ epitaxial thin film has been achieved on *α*-Al_2_O_3_ (0001) substrate by Mn-doping through alternately depositing Ga_2_O_3_ and Mn thin layers using the LMBE technique. The experimental results indicate that Mn ions uniformly distributed in the thin film and replaced Ga sites with an expansion of the crystal lattice. Meanwhile, the prototype photodetector devices with a MSM structure have been fabricated and investigated based on the undoped and Mn-doped Ga_2_O_3_ epitaxial thin films. In compare with the undoped Ga_2_O_3_, the Mn-doped photodetector takes on a lower dark current, a higher sensitivity and a faster photoresponse time, which are attributed to the higher resistivity and a lower trapping states concentration of oxygen vacancies. For the pure Ga_2_O_3_, there are a few oxygen vacancies as donor-type defects leading to the origin of intrinsic n-type conductivity. While for Mn-doping, the extra electrons generated by oxygen vacancies would enter the Mn *d*-shell with the change Mn valence from +3 to +2; that is, the Fermi level would be pinned mid-gap at the Mn 2+/3+ transition level, which is predicted by DFT. The study presents that doping with Mn may provide a convenient and reliable method of obtaining high insulating *β-*Ga_2_O_3_ thin film which is a promising candidate for use in high performance solar-blind photodetector.

## Methods

The thin films were prepared on 10 × 10 mm *α*-Al_2_O_3_ (0001) substrates by the LMBE technique at a repetition frequency of 1 Hz and with a fluence of ~5 J/cm^2^. The thin film deposition was grown in a vacuum environment of 1 × 10^−6^ Pa and at a substrate temperature of 800 °C. For the (GaMn)_2_O_3_ thin film, the alternating depositions of Ga_2_O_3_ and Mn ultrathin layers were performed for 20 times, which enables the realization of Mn-doped Ga_2_O_3_ due to inter diffusion between Mn and Ga_2_O_3_ ultrathin layers at high temperature. The Mn concentrations in (GaMn)_2_O_3_ thin film was determined as 28.4 at.% by XPS. More detail informations about the synthesis, characterizations, photoresponse measurements and simulation calculation can see Supplementary Information.

## Additional Information

**How to cite this article**: Guo, D. *et al.* Inhibition of unintentional extra carriers by Mn valence change for high insulating devices. *Sci. Rep.* **6**, 24190; doi: 10.1038/srep24190 (2016).

## Supplementary Material

Supplementary Information

## Figures and Tables

**Table 1:**
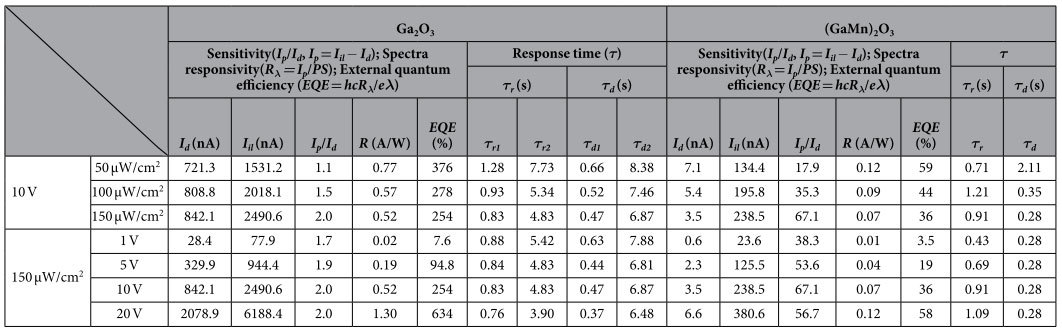
Comparison of the parameters between the Ga_2_O_3_ and (GaMn)_2_O_3_ photodetectors.
