# Supplementary material for: Inhibition of unintentional extra carriers by Mn valence change for high insulating devices
Source: Sci Rep. 2016 Apr 12;6:24190. doi: 10.1038/srep24190 (PMC4828704; doi:10.1038/srep24190)
Supplement: Supplementary Information [file srep24190-s1.pdf]

Supporting Information

Inhibition of unintentional extra carriers by Mn valence change for high insulating devices

Daoyou Guo[1,2], Peigang Li[1,2], Zhenping Wu[1,2], Wei Cui[1,2], Xiaolong Zhao[1,2], Ming Lei[1,2], Linghong Li[3] & Weihua Tang[1,2]

[1] *Laboratory of Optoelectronics Materials and Devices, School of Science, Beijing University of Posts and Telecommunications, Beijing 100876, China.*

[2] *State Key Laboratory of Information Photonics and Optical Communications, Beijing University of Posts and Telecommunications, Beijing 100876, China.*

[3] *Department of Physics, The State University of New York at Potsdam, Potsdam, New York 13676-2294, USA.*

Correspondence and requests for materials should be addressed to W. T. (email: whtang@bupt.edu.cn)

Experiments

Synthesis and Characterizations: For $(GaMn)_2O_3$ thin film, the Mn concentrations can be controlled by solely changing the pulse numbers of the Mn layer during the alternating depositions of Ga_2O_3 and Mn ultrathin layers.[1] Herein, the laser pulse number for Ga_2O_3 and Mn layers were fixed at 100 and 40 in each run. For the pure Ga_2O_3 thin films, the laser pulse number is 2800. The orientation and crystallinity of the as-grown thin films were investigated by XRD at θ-2θ scan and *in-situ* RHEED. For XRD measurement, no impurity peaks related to Mn metal clusters, Mn oxide, or Mn_xGa_y phases were observed in the $(GaMn)_2O_3$ thin film. The surface morphology was characterized by AFM. The thickness and microstructure of thin films were obtained by TEM. Cross-sectional TEM specimens were prepared by a standard procedure which includes mechanical grinding, polishing, precision dimpling, and ion milling. The elements of Ga, Mn, Al, and O were chosen as the target elements for the elemental composition mapping [Fig. 2(b)], which was obtained by the EDX measurement in TEM. The valences of Mn ions and element content were analyzed by XPS.

Fabrication of the Prototype Photodetectors: Radio frequency magnetron sputtering technique was used to deposit four pairs of Au/Ti interdigital electrode on the epitaxial thin films using a shadow mask to construct a MSM photodetector. The schematic diagram of the fabricated prototype photodetector device is shown in Supplementary Fig. S1. The electrode fingers were 100 μm wide, 2800 μm long, and

100 μm spacing gap. And the effective irradiated area was ~ 0.021 cm^2.

Photoresponse Measurements: The *I-V* characteristics and time-dependent photoresponse of photodetectors were measured by Keithely 2450. An ultraviolet lamp with a wavelength of 254 nm was as the light source.

Simulation calculation: The calculations of the electronic structure of undoped and Mn-doped β-Ga_2O_3 were completed by CASTEP software package in Materials Studio (MS) 4.0 software.[2] It is a quantum mechanics program *ab initio* calculation based on DFT. The plane wave ultra-soft pseudo-potential was selected to describe the interaction between the electron and the ionic cores. The Perdew-Burke-Ernzerhof (PBE) scheme of the generalized gradient approximation (GGA) was adopted for describing the exchange correlation interactions.

Results and Discussion

For the epitaxial relationship between $(\bar{2}01)$ β-Ga_2O_3 and (0001) α-Al_2O_3, the oxygen atoms in the $(\bar{2}01)$ equivalent plane of β-Ga_2O_3 have the same arrangement as the oxygen atoms of the Al_2O_3 (0001) plane.[3] Moreover, 4 lattice planes of β-Ga_2O_3 in the [010] direction match very closely with 3 lattice planes of Al_2O_3 in the $[01\bar{1}0]$ direction, and 2 lattice planes of β-Ga_2O_3 in the [201] direction match very closely with 3 lattice planes of Al_2O_3 in the $[2\bar{1}\bar{1}0]$ direction.[4] The lattice mismatches between the thin film and substrate are -1.6% and +3.13% respectively, which is accommodated by the domain variation principle.[5] Therefore, when β-Ga_2O_3 is grown on the (0001) α-Al_2O_3 substrate, gallium will bond

to the oxygen atom layer almost without showing the difference between the (0001) α-Al_2O_3 and the $(\bar{2}01)$ plane of β-Ga_2O_3.

For the parameters of photodetector, the sensitivity of the photodetector, defined as $(I_{il}$-$I_d)/I_d$ in percent (I_{il} is the current of the device when illuminated with a light source and I_d is the dark current), the spectra responsivity (R_λ), defined as the photocurrent generated per unit power of incident light on the effective area of a photoconductor, and the external quantum efficiency (*EQE*), defined as the number of electrons detected per incident photon, are the key parameters to evaluate the performance of a photodetector.[6-8] The larger values of sensitivity, R_λ and *EQE* – the higher performance a photodetector has. The R_λ and *EQE* can be calculated in the following equations: $R_\lambda = I_p/PS$ and $EQE = hcR_\lambda/e\lambda$, where the photocurrent is the difference between an illuminated and a dark current ($I_p = I_{il} - I_d$), P is the light intensity illuminated on the devices, and S is the effective illuminated area, h is Planck's constant, c is the velocity of light, e is the electronic charge, and λ is the wavelength of illuminating light.[6] The quantitative analysis of the current rise and decay process involves the fitting of the photoresponse curve with a bi-exponential relaxation equation of the following type: $I = I_0 + Ae^{-t/\tau_1} + Be^{-t/\tau_2}$, where I_0 is the steady state photocurrent, t is the time, A and B are constant, τ_1 and τ_2 are two relaxation time constants.[9] For the time-dependent photoresponse, the devices still exhibits a nearly identical response after multiple illumination cycles, indicating the high robustness and good reproducibility of the photodetectors.

For the conventional unit cell of β-Ga_2O_3, it belongs to a monoclinic lattice (the space group C/2m) with the unit cell parameters: a =1.223 nm, b = 0.304 nm, c = 0.580 nm, and β = 103.7.[10] There are four Ga_2O_3 in one unit cell and two kinds of coordination for Ga^{3+} ions in this structure, namely tetrahedral and octahedral. The doubly connected straight chains of GaO_6 edge shared octahedra run along b, and the chains are connected by GaO_4 tetrahedra to one another.

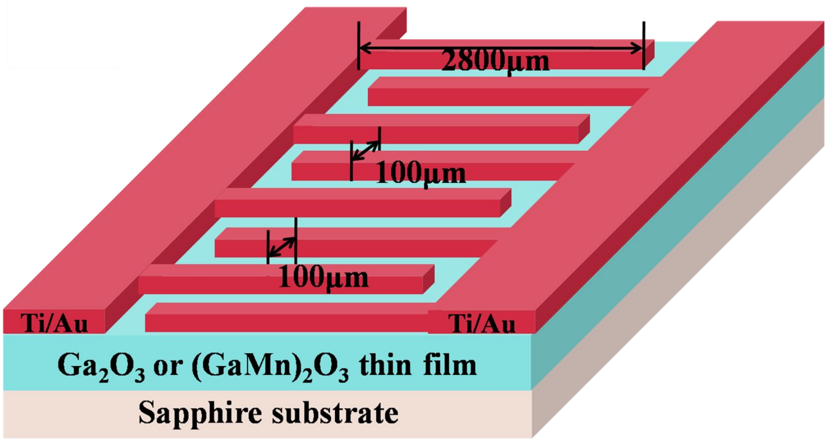

Supplementary Figure S1. Schematic diagram of the MSM structure photodetector.

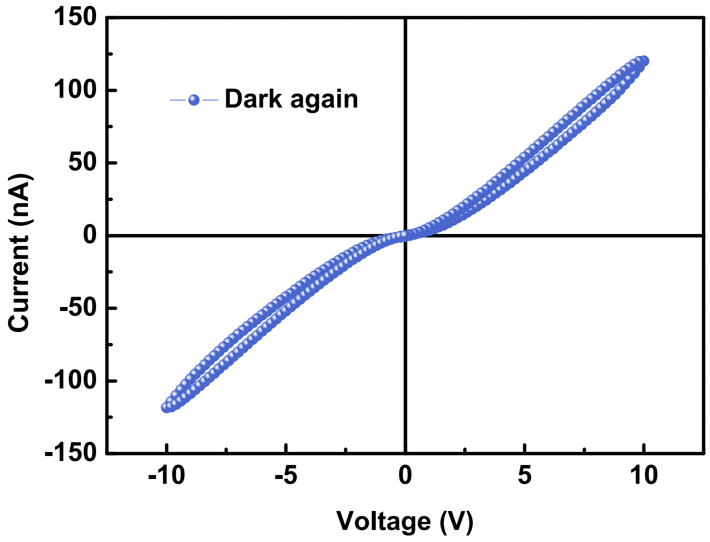

Supplementary Figure S2. The dark *I-V* characteristic curve of the Ga_2O_3 photodetector after turning off the 254 nm light for 24 hours.

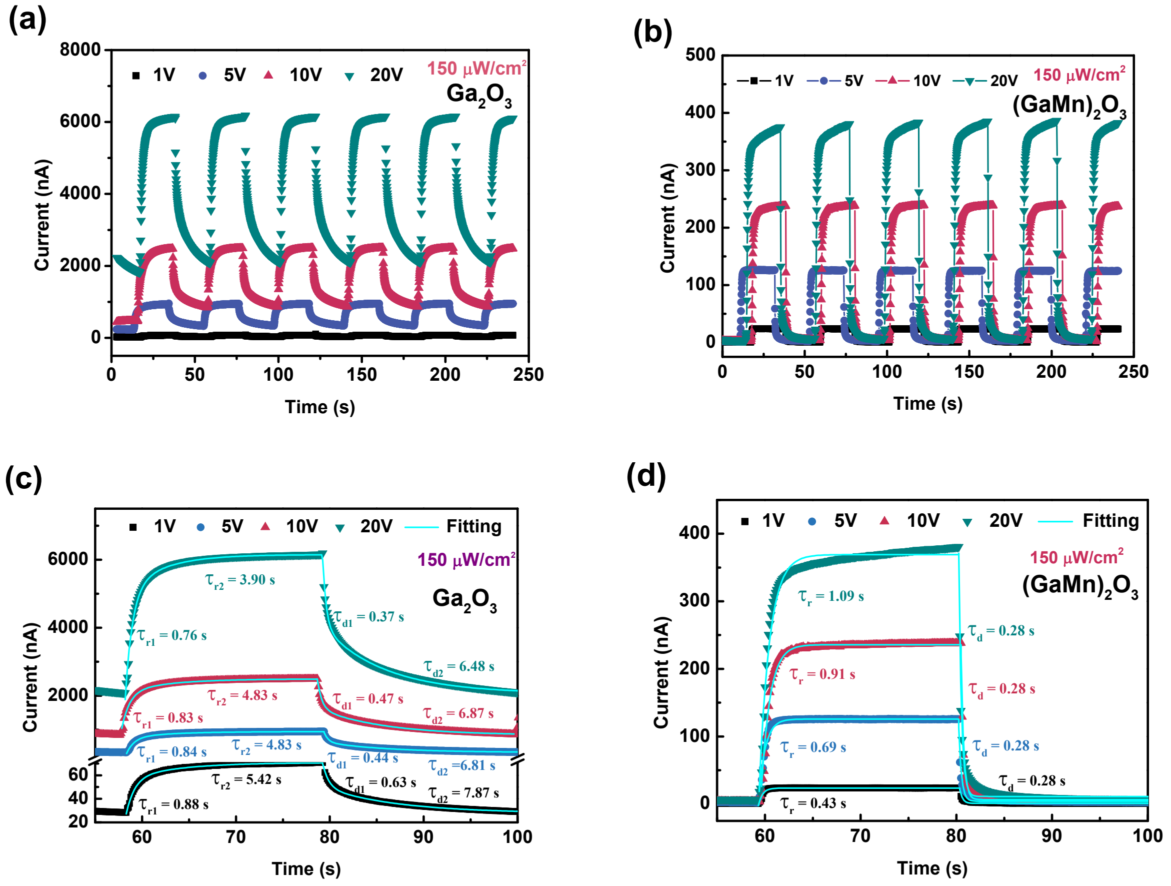

Supplementary Figure S3. Time-dependent photoresponse of the Ga_2O_3 (a) and $(GaMn)_2O_3$ (b) photodetector to 254 nm light illumination with an optical input power of 150 μW/cm^2 by on/off switching under varied applied bias (1, 5, 10, 20 V), and (c), (d) are the corresponding fitted curve for the current rise and decay process of (a), (b) respectively.

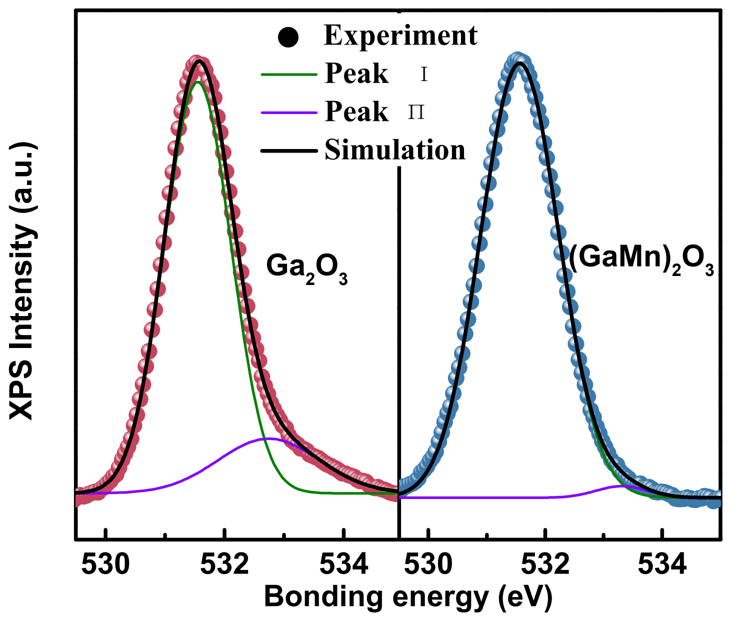

Supplementary Figure S4. O 1s XPS spectra of the Ga_2O_3 (left) [11] and $(GaMn)_2O_3$ (right) thin films.

References

1. Guo, D. Y. *et al.* Room temperature ferromagnetism in $(Ga_{1-x}Mn_x)_2O_3$ epitaxial thin films. *J. Mater. Chem. C* **3**, 1830 (2015).

2. Segall, M. D. *et al.* First-principles simulation: ideas, illustrations and the CASTEP code. *J. Phys.: Condens. Matter.* **14**, 2717 (2015).

3. Nakagomi, S. *et al.* Crystal orientation of β-Ga_2O_3 thin films formed on c-plane and a-plane sapphire substrate. *J. Cryst. Growth* **349**, 12 (2012).

4. Ohta, H. *et al.* Epitaxial growth of transparent conductive oxides. *Int. J. Mod. Phys. B* **16**, 173 (2002).

5. Narayan, J. *et al.* Domain epitaxy: A unified paradigm for thin film growth. *J. Appl. Phys.* **93**, 278 (2003).

6. Tian, W. *et al.* In-doped Ga_2O_3 nanobelt based photodetector with high sensitivity and wide-range photoresponse. *J. Mater. Chem.* **22**, 17984 (2012).

7. Wu, J. M. *et al.* Ultrahigh responsivity and external quantum efficiency of an ultraviolet-light photodetector based on a single VO_2 microwire. *ACS Appl. Mater. Interfaces* **6**, 14286 (2014).

8. Zheng, W. *et al.* low-dimensional structure vacuum-ultraviolet-sensitive ($\lambda < 200$ nm) photodetector with fast-response speed based on high-quality AlN micro/nanowire. *Adv. Mater.* **27**, 3921 (2015).

9. Liu, N. *et al.* Direct growth of lateral ZnO nanorod UV photodetectors with Schottky contact by a single-step hydrothermal reaction. *ACS Appl. Mater. Interfaces* **2**, 1973 (2010).

10. Bermudez, V. M. The structure of low-index surfaces of β-Ga_2O_3. *Chem. Phys.* **323**, 193 (2006).

11. Guo, D. Y. *et al.* Oxygen vacancy tuned Ohmic-Schottky conversion for enhanced performance in β-Ga_2O_3 solar-blind ultraviolet photodetectors, *Appl. Phys. Lett.* **105**, 023507 (2014).
